# Migraine attacks are of peripheral origin: the debate goes on

**DOI:** 10.1186/s10194-022-01538-1

**Published:** 2023-01-10

**Authors:** Thien Phu Do, Anders Hougaard, Greg Dussor, K. C. Brennan, Faisal Mohammad Amin

**Affiliations:** 1 Danish Headache Center, Department of Neurology, Rigshospitalet Glostrup, Faculty of Health and Medical Sciences, University of Copenhagen, Copenhagen, Denmark; 2 School of Behavioral and Brain Sciences, Center for Advanced Pain Studies, University of Texas at Dallas, Richardson, TX 75080 USA; 3 Department of Neurology, Albert Einstein College of Medicine, 1300 Morris Park Avenue, Bronx, NY 10461 USA; 4 Department of Neurorehabilitation/Traumatic Brain Injury, Rigshospitalet, University of Copenhagen, Copenhagen, Denmark

**Keywords:** Headache, Migraine with aura, Migraine without aura, Meningeal artery, Peripheral, Central, Origin, Premonitory, Prodromal, Cortical spreading depression, Nociception, Human models

## Abstract

**Background:**

Despite the pervasiveness of migraine, the underlying pathophysiological mechanisms initiating migraine attacks are far from well understood and are matter of scientific debate.

**Objective:**

In this narrative review, we discuss key evidence for that suggest a peripheral origin or central origin and provide directions for future studies that may provide further clarification.

**Discussion:**

Migraine pathogenesis is considered to involve the trigeminovascular system, a term that encompasses the trigeminal nerve and its axonal projections to the intracranial blood vessels. Beyond any doubt both peripheral and central mechanisms are involved in migraine pathogenesis, but an unresolved question is the how the initial activation occurs in a migraine attack. Evidence favoring a peripheral origin of migraine attacks, i.e., initial events occur outside of the blood–brain barrier, include the importance of sensitization of perivascular sensory afferents early on in a migraine attack. Evidence favoring a central origin include the occurrence of prodromal symptoms, migraine aura, and activation of structures within the central nervous system early in and during a migraine attack.

**Conclusions:**

Both peripheral and central mechanisms are likely involved in a migraine attack, e.g., peripheral nociceptive input is necessary for pain transmission and cortical activity is necessary for pain perception. Yet, the debate of whether migraine attacks are initiated a peripheral or central site remains unresolved. The increased focus on prodromal symptoms and on the development of a human model of migraine aura will possibly provide key arguments needed to answer this question in the near future. Until then, we cannot draw firm conclusions and the debate goes on.

**Video link:**

Video recording of the debate held at the 1st International Conference on Advances in Migraine Sciences (ICAMS 2022, Copenhagen, Denmark) is available at: https://www.youtube.com/watch?v=NC0nlcKohz0.

**Graphical Abstract:**

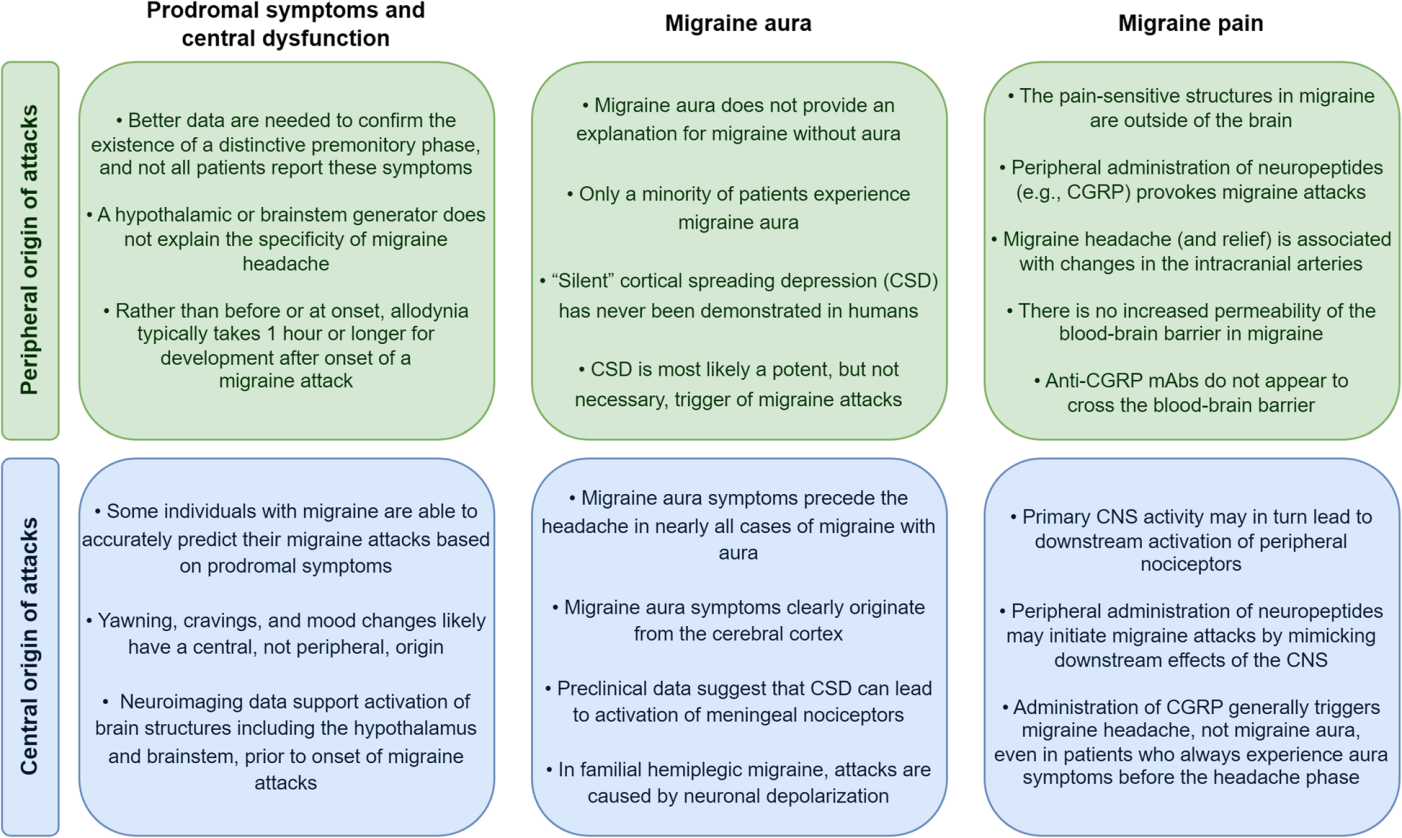

## Introduction

Migraine is a common neurological disorder characterized by recurrent headache attacks of moderate-to-severe pain intensity accompanied by a range of symptoms including nausea, vomiting and hypersensitivity to light and sounds [1, 2]. Despite its pervasiveness, the underlying pathophysiological mechanisms initiating migraine attacks are far from well understood and are matter of scientific debate. On one side, evidence favoring a peripheral origin of migraine attacks, i.e., initial events occur outside of the blood–brain barrier, include the importance of sensitization of perivascular sensory afferents early on in a migraine attack [3, 4], and that migraine attacks can be triggered and attenuated using pharmacological compounds that do not appear to need to cross the blood–brain barrier to exert their effects [5]. In contrast, occurrence of prodromal symptoms, i.e., a symptoms before the onset of headache, migraine aura, and data supporting dysfunction of the diencephalon and the brainstem suggest a migraine attack generator localized within the central nervous system [3, 4]. In this narrative review, we will describe some of the key evidence that suggest a peripheral origin or a central origin of migraine attacks, respectively. In particular, we focus on the possible role of prodromal symptoms and central dysfunction, migraine aura, and migraine pain (Table 1).

**Table 1:** Overview of key arguments for a peripheral or central origin of migraine attacks

	**Peripheral Origin**	**Central Origin**
**Prodromal symptoms and central dysfunction**	• Methodological uncertainties limits inferences from reports on prodromal symptoms in migraine • The hypothesis of a hypothalamic or brainstem generator of migraine attacks does not explain specificity to migraine headache in humans • Rather than before or at onset, allodynia typically takes 1 h or longer for development after onset of a migraine attack	• Some individuals with migraine are able to accurately predict their migraine attacks based on prodromal symptoms • Common migraine prodromal symptoms such as yawning, cravings, and mood changes likely have a central, not peripheral, origin • Advanced neuroimaging studies support activation of brain structures including the hypothalamus and brainstem, prior to onset of migraine attacks
**Migraine aura**	• Migraine aura does not provide an explanation for the most common phenotype, migraine without aura • Migraine aura is not a prerequisite of migraine attacks. Only a minority of patients experience migraine aura • “Silent” cortical spreading depression has never been demonstrated in humans • Observations of migraine aura without headache suggests that cortical spreading depression is but a potent potential trigger of migraine attacks without aura • Symptoms in monogenic subtypes of migraine (e.g., hemiplegia during aura, progressive ataxia, attacks triggered by mild head trauma; brain edema, mental retardation, and progressive ataxia) are not found in common migraine subtypes	• Migraine aura symptoms precede the headache in nearly all cases of migraine with aura • Migraine aura symptoms clearly originate from the cerebral cortex • Preclinical studies have demonstrated that cortical spreading depression, the underlying mechanism of aura, can lead to activation of meningeal nociceptors • In familial hemiplegic migraine, attack initiation is understood on the molecular level and is explained by ion transport dysfunction leading to neuronal depolarization • A recent meta-analysis of genetic studies supports that migraine with typical aura may involve dysfunction of similar ion transporters in the cerebral cortex
**Migraine pain**	• Pain-sensitive structures are limited to the surrounding structures of the brain, e.g., dura mater, pia mater and their feeding vessels, and not the brain parenchyma itself • Peripheral administration of neuropeptides, e.g., calcitonin gene-related peptide (CGRP), provokes migraine attacks in susceptible individuals. These compounds do not cross the blood–brain barrier • There is no increased permeability of the blood–brain barrier in individuals with migraine without aura or migraine with aura • Migraine headache (and relief) is associated with changes in the circumference of intracranial arteries • Monoclonal antibodies targeting the CGRP signaling pathway are highly effective for migraine prevention and these molecules do not cross the blood–brain barrier • Epidemiological (association with cardiovascular disease) and genetic data suggest an association between migraine and vascular dysfunction	• Although the pain perceived during migraine attacks may originate peripheral structures, the primary events of migraine may well occur in the central nervous system (CNS), as suggested by the early symptoms of migraine, i.e., prodromal symptoms and migraine aura • In spontaneous migraine attacks, primary CNS activity may in turn lead to downstream activation of peripheral nociceptors • Hypothetically, peripheral administration of neuropeptides may initiate migraine attacks by mimicking downstream effects of the CNS in spontaneous migraine attacks. Administration of CGRP generally triggers migraine headache, not migraine aura, even in patients who always experience aura symptoms before the headache phase in the course of their spontaneous migraine attacks • Genetic data suggest an association between migraine and central dysfunction

*CGRP* Calcitonin gene-related peptide, *CNS* Central nervous system

## Search strategy and selection criteria

We searched MEDLINE (in both cases from database inception to August 1, 2022) for original research articles, systematic reviews and meta-analyses. We used the search term “migraine” in combination with the terms “epidemiology”, “pathophysiology”, “premonitory”, “prodromal”, “aura”, “central dysfunction”, “nociception”, “diagnosis” and/or “treatment”. We preferentially selected publications from the past 10 years but did not exclude commonly referenced and highly regarded older publications. We also searched the reference lists of articles identified by this search strategy and selected those we judged relevant.

## Migraine attacks are of peripheral origin

### Prodromal symptoms and central dysfunction

Individuals with migraine may report a symptomatic phase before the onset of pain in migraine [6, 7], so-called prodromal symptoms (previously known as premonitory symptoms). Although not all patients report prodromal symptoms, understanding the underlying mechanisms of this proposed prodromal symptomatic phase in migraine may provide insights into the mechanisms of migraine attack initiation [1, 2, 7]. Yet, estimates of the relative frequency of prodromal symptoms fluctuate considerably between epidemiological studies [7], and it remains unclear if any specific individual symptoms are characteristic of this proposed phase [8]. Non-specific symptoms, e.g., fatigue, irritability, food cravings, yawning, are among the most frequently reported symptoms before onset of a migraine attack [7], which may suggest limbic dysfunction (e.g., dopaminergic and hypothalamic dysfunction). However, these are common symptoms amongst both individuals without and with migraine, and often have no association with a migraine attack [9]. These uncertainties question whether the available evidence can confirm the existence of a prodromal phase as a distinct component of a migraine attack. As patients can have migraine attacks without reporting prodromal symptoms, this suggests that the underlying mechanisms are not necessary to develop a migraine attack, but rather, they may co-occur as epiphenomena. A key feature of migraine is that attacks can be provoked, which provides a framework for investigating migraine pathophysiology by deliberately triggering migraine attacks in humans, i.e., a human provocation model [5]. Interestingly, this model has demonstrated that intravenous administration of calcitonin gene-related peptide (CGRP) and pituitary adenylate cyclase-activating peptide (PACAP) to susceptible individuals can induce migraine attacks [10, 11]. However, these migraine attacks occurred without prodromal symptoms in most cases following administration of CGRP and PACAP in a clinical trial [12]. Moreover, in those individuals who did report prodromal symptoms, these sometimes occur at or after onset of headache – or without any migraine attack developing at all [12].

Furthermore, the hypothesis of a hypothalamic or brainstem generator of migraine attacks does not explain specificity to migraine headache in humans and exclusion of other nociceptive dorsal horn neurons [4]. Most likely, brainstem activation as observed on neuroimaging studies in humans is dependent on activation of meningeal or other intracranial nociceptors [4]. Of note, several neuroimaging studies reporting activation of central structures, e.g., brainstem, were reliant on peripheral noxious stimulation or human provocation model with a peripheral administration of a pharmacological trigger [13]. Interestingly, activation of the periaqueductal grey matter is a consequence of nociceptor activation anywhere in the body; it is highly unspecific and involve areas outside the trigeminal system and cannot be used as a marker for a pain phenomenon restricted exclusively to trigeminal regions [14–17]. In clinical case reports of headache secondary to brainstem lesions, it cannot be excluded that there was direct or indirect activation of intracranial nociceptors due to close proximity, and the phenotype of these headaches rarely resemble migraine-like pain [18–20].

Another line of reasoning for a central sensitization/dysfunction is the occurrence of allodynia in individuals with migraine [21, 22]. One would expect that allodynia would develop before the onset of a migraine attack if central sensitization/dysfunction is the etiology, but allodynia typically takes one hour or longer for development after onset of a spontaneous migraine attack [23]. Furthermore, one-third of individuals with migraine do not experience allodynia as an accompanying symptom of migraine attacks [21, 22]. More likely, central sensitization in migraine is driven by a nociceptive peripheral input [4].

### Migraine aura

While migraine aura clearly has a cortical origin with cortical spreading depression (CSD) as the underlying neurobiological mechanism [24], this is not a prerequisite for migraine headache. Migraine aura is only experienced by one-third of individuals with migraine [25], and in some of these patients, migraine aura does not occur consistently through all attacks. In addition, headache, as well as other migraine-associated symptoms, are present early during the aura phase in most patients, and sometimes even before the aura phase [26]. More importantly, migraine aura does not provide an explanation for migraine attacks without aura. Occurrence of “silent” migraine aura, i.e., an event of CSD without focal neurological symptoms, has been proposed to occur prior to migraine attacks without aura. However, this has never been demonstrated in humans and remains speculative. Hemodynamic changes associated with CSD has been described on neuroimaging in individuals experiencing migraine aura [27, 28], but these findings cannot be reproduced in studies of individuals with migraine attacks without aura [29]. Furthermore, observations of individuals who experience migraine aura without headache suggests that cortical spreading depression is merely another potential trigger of a migraine headache [30–33]. This is emphasized through human provocation studies of patients with migraine with aura, who report a migraine attack without aura for the first time in their lives following peripheral administration of an experimental trigger compound, e.g., CGRP [5, 34]. As CGRP is not able to cross the blood–brain barrier [35], and current evidence suggest there is no blood–brain barrier disruption in migraine pathophysiology [36, 37], the site of action is likely outside of the brain. Interestingly, there are reports of migraine aura following peripheral administration of CGRP [5, 34], and migraine aura-like phenomena and CSD can be caused by vascular events, e.g., carotid dissection, arteriovenous malformations [38, 39]. Whether CSD in these cases is caused by reasons such as microembolization, focal ischemia through disruption in blood flow or disturbances in local homeostasis secondary to these events remains speculative, but fact is that symptomatic migraine with aura can occur due to an initial vascular circumstance [40].

In vivo studies of mice with knock-ins of two different familial hemiplegic migraine (FHM)-genes showed increased susceptibility to and propagation velocity of cortical spreading depression compared with wild-type animals [41–44]. However, these findings are not necessarily relevant for other migraine types [45–48], and many of the traits found in these monogenic subtypes of migraine (e.g., hemiplegia during aura, progressive ataxia, attacks triggered by mild head trauma; brain edema, mental retardation, and progressive ataxia) are certainly not found in common migraine subtypes.

Based on these observations, migraine aura and cortical spreading depression are likely another potential trigger of migraine headache and does not provide an explanation for the most common phenotype, migraine attacks without aura.

### Migraine pain

The most convincing arguments for a peripheral origin of migraine attacks is the fact that migraine attacks can be produced and attenuated entirely at peripheral sites of action. A key feature of migraine is that various trigger factors are able to provoke migraine attacks. Human provocation models draw advantage of this feature, wherein endogenous molecules or other putative triggers are administered in humans to induce migraine, to identify signaling pathways that are involved in migraine pathophysiology. Series of randomized trials with human provocation models have consistently demonstrated that intravenous administration of various neuropeptides, e.g., CGRP and PACAP [49–51], is able to induce migraine attacks in susceptible individuals [5]. The site of action of these neuropeptides (e.g., CGRP and PACAP) includes the cranial arteries as neuroimaging studies consistently demonstrate a marked extracerebral vasodilation but not of the large cerebral arteries following administration [2, 5, 11]. Interestingly, animal models demonstrate that administration of these n CGRP and PACAP within the central nervous system can induce antinociception rather than nociception [52–55]. Observations during neurosurgical procedures in awake patients suggest that the pain-sensitive structures are limited to the surrounding structures of the brain, e.g., dura mater, pia mater and their feeding vessels, whereas stimulation of the brain parenchyma itself does not evoke pain [56]. The trigeminovascular system provides a framework wherein peripheral input can lead to migraine attacks through sensitization and activation of trigeminal primary afferents, mediated through vasodilation of intracranial arteries [2, 3]. A proposed mechanism of how this vascular signaling contributes to pain perception involves sensitization through increased extracellular potassium [2]. Arterial vasodilation is caused by opening of cation channels, mainly potassium channels, in vascular smooth muscle cells and results in accumulation of positively charged ions in the extracellular space [2]. In turn, this electrical gradient drives positively charged ions into and activate neighboring trigeminal pain fibers [2]. This is supported by the observation that activation of downstream targets of CGRP and PACAP, e.g., K_ATP_ channels and BK_Ca_ channels, induces migraine attacks at a much higher rate in parallel with a marked vasodilation, which suggests a vascular site of action [57, 58]. Interestingly, while these channels are also expressed in C- and Aδ-fibers, intradermal and intramuscular injections of levcromakalim, a K_ATP_ channel opener, does not evoke cutaneous or muscle pain [59]; in turn, a direct activation of these channels in peripheral neurons is an unlikely site of action for migraine attacks.

In other paroxysmal pain disorders, e.g., familial episodic pain syndrome, ion channels have been demonstrated to exhibit modulatory activity and provides a context for the episodic nature of migraine attacks [60, 61]. In line with these observations, a genome wide association meta-analysis identified 123 susceptibility loci that showed enrichment for genes expressed in vascular and smooth muscle tissues in individuals with migraine without or with aura [62]. These findings suggest vascular dysfunction, and possibly also smooth muscle dysfunction (consistent with a shared polygenic risk scores of migraine, stroke, and cardiovascular diseases) [63–67], are crucial in migraine pathogenesis and strongly implicates a vascular etiology of migraine. Interestingly, rare vasculopathies have an overrepresentation of migraine. Cerebral autosomal dominant arteriopathy with subcortical infarcts and leukoencephalopathy (CADASIL) is a hereditary small artery disease and one of its characteristic presentations include migraine [68]. Patients with mitochondrial encephalopathy, lactic acidosis and stroke-like episodes (MELAS) are especially known to suffer from repeated episodes of migraine [69]. Morphological observations suggest MELAS involves mitochondrial angiopathy as autopsies of patients show abnormally proliferated mitochondria in the smooth muscle cells and endothelial cells of the small cerebral blood vessels [70].

Migraine drugs that purely exhibit their effects at a peripheral site of action exist, i.e., monoclonal antibodies targeting CGRP or its receptor, as they are very unlikely to cross the blood brain barrier [35, 71]. Interestingly, these monoclonal antibodies are also effective in patients with migraine with aura [72, 73]. Other compounds with a predominant peripheral site of action include onabotulinumtoxinA where the mechanism of action is likely to involve attenuation of peripheral pain transmission [74]. In individuals with chronic headache, the clinical effect of onabotulinumtoxinA has been suggested to involve reduction in periosteal inflammation [75]. These findings are consistent with the observation that onabotulinumtoxinA is able to reduce release of inflammatory and excitatory neurotransmitters and neuropeptides from primary nociceptors [76, 77]. Of note, adverse events related to the central nervous system are not reported for either monoclonal antibodies nor onabotulinumtoxinA [71]. These observations are also true for commonly used acute migraine medications. Dihydroergotamine (acute migraine drug) is not able to cross the blood–brain barrier in humans [78], and while sumatriptan (acute migraine drug) is able to cross the blood–brain barrier and may present with adverse events related to the central nervous system, preclinical models suggest the site of action for its migraine-attenuating effects is likely mediated through modulation of the first-order neuron localized outside of the blood–brain barrier [79]. This is supported by the observation that sumatriptan constricts the superficial temporal artery and middle meningeal artery, but not the middle cerebral artery, in migraine attacks in humans, which favors a perivascular site of action outside of the blood–brain barrier [80].

## Migraine attacks are of central origin

### Prodromal symptoms and central dysfunction

From a clinical perspective, the site of origin of a migraine attack is reflected by the earliest symptoms of the attack. Many migraine patients report prodromal symptoms, which include mood changes, excessive yawning, thirst, and cravings for certain foods [81]. Such symptoms would be expected to arise from brain regions such as the hypothalamus or other parts of the limbic system, and not from peripheral nerves or from blood vessels. The symptoms may not be specific for migraine but, importantly, a prospective study demonstrated that some patients can accurately predict their migraine attacks based on these early symptoms indicating that prodromal symptoms are truly linked to migraine at least in a subgroup of patients [82].

The notion that this early phase of the migraine attack originates in the brain is supported by a line of evidence based on advanced neuroimaging studies. One study investigated migraine patients during glyceryl trinitrate-induced prodromal symptoms and subsequent migraine headache and found increased activity of the hypothalamus, brainstem, and various cortical areas specifically during the prodromal phase [83]. Another study using BOLD functional MRI and painful trigeminal stimulation to study a migraine patient every day for 30 days, and during three spontaneous migraine attacks, found increased activation of the hypothalamus, and increased functional connectivity between the hypothalamus and the pons within 24 h before headache onset [13]. The authors later reproduced these findings of preictal hypothalamic activation in seven migraine patients scanned every day for at least 30 consecutive days [84].

### Migraine aura

Approximately one-third of migraine patients experience aura symptoms. The aura symptoms begin before the onset of pain in nearly all cases [85] and based on their clinical presentation they clearly originate from the cerebral cortex [86]. The underlying mechanism of migraine aura is widely accepted to be the electrophysiological phenomenon of CSD, involving a wave of neuronal and glial depolarization spreading across the cerebral cortex at an approximate rate of 3 mm/min [87]. Although electrophysiological recordings from the cortical surface have not been performed during attacks of migraine with aura, gradually spreading changes of cerebral blood flow consistently shown in functional neuroimaging studies support that the aura phase of migraine is indeed due to CSD [28, 88]. Interestingly, a recent PET-MRI study in migraine aura patients, applying a radioactive marker of inflammation, indicated that CSD may directly induce meningeal inflammation and thereby potentially head pain and associated symptoms of migraine [89]. Thus, CSD appears to be a primary event that precedes, and causes, the pain phase of migraine. In support of this, animal studies have demonstrated that experimentally induced CSD leads to activation of meningeal nociceptors and central trigeminovascular neurons [90]. In addition, CSD leads to pain and anxiety behavior in animals even when elicited in a minimally invasive manner using optogenetics [91].

Collectively, migraine attacks with aura clearly originate from the cerebral cortex. In the subset of patients with familial hemiplegic migraine (FHM), the site of origin can even be specified at the molecular level. Three genes are known to be involved in familial hemiplegic migraine. In FHM type 1, mutations in CACNA1A, encoding the α1 subunit of the voltage-gated channel CaV2.1 calcium leads to gain of channel function. FHM type 2 mutations in the ATP1A2 gene encoding the α2 subunit of Na/K-ATPases result in a loss of function in glial cells. In FHM type 3, mutations in the SCN1A gene lead to a gain of function of NaV1.1 sodium channels. These mutations facilitate neuronal depolarization. Knock-in mouse models have been developed for all three types of familial hemiplegic migraine and in these an enhanced susceptibility to experimental CSD elicitation has been demonstrated [92]. Interestingly, a recent genome-wide analysis of 102,084 migraine cases indicated that the CACNA1A gene is also involved in migraine with typical aura [62]. Likely, migraine with aura patients in general are susceptible to CSD initiation due to ion channel dysfunction leading to occasional neuronal depolarization.

Attacks of symptoms that are clinically indistinguishable from migraine with aura may occur due to e.g., carotid dissection or carotid aneurysms [40]. The likely mechanism behind this observation is that carotid pathology may lead to hypoperfusion or microembolization, which is known to be able to trigger CSD [93]. Clear-cut cases of “symptomatic migraine aura”, although apparently rare, have been reported and are most often caused by lesions to the cerebral cortex including brain arteriovenous malformations or brain tumors [40]. Rare vasculopathies including CADASIL, MELAS, Sneddon syndrome, and Moyamoya disease may present with migraine with aura as well as stroke [94]. In these cases, attacks of migraine with aura are likely secondary to cortical lesions caused by the cerebrovascular pathology.

### Migraine pain

It is possible that the pain of migraine originates from peripheral structures although there is no firm evidence to support this. Vasodilation seems not be the cause of pain in migraine. Even strong vasodilation of cephalic arteries causes only mild headache [95] and there is no correlation between the degree of vasodilation and pharmacologically induced headache in healthy volunteers [96]. An MR angiography study of spontaneous migraine attacks without aura reported slight dilation of intracranial, but not extracranial, arteries during attacks [97]. Administration of subcutaneous sumatriptan resulted in pain relief but not constriction of the dilated intracerebral arteries. Triptans, 5-HT_1B/1D_ agonists cross the blood–brain barrier [98] and their anti-migraine effects may depend on binding to central serotonin receptors. Likewise, ditans, i.e., 5-HT_1F_ agonists, cause relief of migraine headache, cross the blood–brain barrier, and do not appear to cause vasoconstriction [99, 100]. Even a central action of monoclonal antibodies cannot be excluded with certainty since these may cross the blood–brain barrier although at a small rate of 1:1000 [101].

Migraine-inducing neuropeptides like CGRP may exert their effects in the periphery but this does not provide evidence of a peripheral origin of spontaneous migraine attacks. Interestingly, drugs that provoke migraine attacks clinically generally do not cause migraine aura symptoms, even in patients who always experience aura during their spontaneous migraine attacks [5]. Thus, these substances likely exert their migraine-provoking effects peripherally and downstream as opposed to spontaneous migraine attacks that originate centrally and subsequently lead to peripheral effects.

## Lessons learned and future directions

### Prodromal symptoms and central dysfunction

Methodological uncertainties limits inferences from reports on prodromal symptoms in migraine, which otherwise may assist our understanding of migraine attack initiation. Heterogeneity between investigations, including application of different definitions and matters of enquiry, allows for large discrepancies. Furthermore, non-specific symptoms such as fatigue and mood change are commonly reported, and strict criteria need to be applied to allow discrimination between spontaneous occurrence or migraine-associated occurrence. These shortcomings can be addressed through harmonization of studies by standardized methodology and data reporting. The fact there are no internationally acknowledged guidelines yet warrants an investment.

### Migraine aura

Although migraine aura and migraine headache are temporally associated in many cases, it may be counter-productive to discuss these disorders as a single entity as their mechanistic relationship is not clarified. Experimental research in humans has been limited by the lack of a potent human model of migraine aura. Recent findings suggest that opening of potassium channels may be a potent trigger of migraine aura in humans [102]. If these observations are confirmed, it would allow for investigations into the mechanisms that link migraine aura and migraine headache using neuroimaging, electrophysiology, and biochemistry.

### Migraine pain

Beyond any doubt, migraine pain is modulated through activity of CGRP and other neuropeptides within the trigeminovascular system [3]. Release of CGRP and other neuropeptides have been demonstrated to be released at a peripheral site at the level of the trigeminal ganglion [103], but we cannot at the present time confirm or reject a downstream regulation through central dysfunction as there is an absence of evidence, not evidence of absence. While cortical spreading depression is able to depolarize meningeal nociceptors [24], thereby causing pain, this does not provide an explanation for the majority of migraine attacks experienced in the world: migraine attacks without aura. Experimental investigations need to address whether it is possible to induce a peripheral release of these neuropeptides through a central mechanism in humans with migraine without aura.

## Conclusions

Both peripheral and central mechanisms are likely involved in a migraine attack, e.g., peripheral nociceptive input is necessary for pain transmission and cortical activity is necessary for pain perception. Yet, the debate of whether migraine attacks are initiated a peripheral or central site remains unresolved. The increased focus on prodromal symptoms and on the development of a human model of migraine aura will possibly provide key arguments needed to answer this question in the near future. Until then, we cannot draw firm conclusions and the debate goes on.

## Data Availability

No data were generated for this manuscript.

## References

[1] Ashina M, Terwindt GM, Al-Karagholi MA-M (2021). Migraine: disease characterisation, biomarkers, and precision medicine. Lancet.

[2] Ashina M (2020). Migraine. N Engl J Med.

[3] Ashina M, Hansen JM, Do TP (2019). Migraine and the trigeminovascular system—40 years and counting. Lancet Neurol.

[4] Olesen J, Burstein R, Ashina M (2009). Origin of pain in migraine: evidence for peripheral sensitisation. Lancet Neurol.

[5] Ashina M, Hansen JM, Á Dunga BO (2017). Human models of migraine-short-Term pain for long-Term gain. Nat Rev Neurol.

[6] Headache Classification Committee of the International Headache Society (IHS) (2018) The International Classification of Headache Disorders, 3rd edition. Cephalalgia 38(1):1–211. 10.1177/0333102417738202 10.1177/0333102417738202 29368949

[7] Karsan N, Goadsby PJ (2018). Biological insights from the premonitory symptoms of migraine. Nat Rev Neurol.

[8] Eigenbrodt AK, Christensen RH, Ashina H (2022). Premonitory symptoms in migraine: a systematic review and meta-analysis of observational studies reporting prevalence or relative frequency. J Headache Pain.

[9] Ricci JA, Chee E, Lorandeau AL (2007). Fatigue in the U.S. Workforce: Prevalence and Implications for Lost Productive Work Time. J Occup Environ Med.

[10] Lassen L, Haderslev P, Jacobsen V (2002). Cgrp May Play A Causative Role in Migraine. Cephalalgia.

[11] Schytz HW, Birk S, Wienecke T (2009). PACAP38 induces migraine-like attacks in patients with migraine without aura. Brain.

[12] Guo S, Vollesen ALH, Olesen J (2016). Premonitory and nonheadache symptoms induced by CGRP and PACAP38 in patients with migraine. Pain.

[13] Schulte LH, May A (2016). The migraine generator revisited: continuous scanning of the migraine cycle over 30 days and three spontaneous attacks. Brain.

[14] Derbyshire SWG, Jones AKP, Creed F (2002). Cerebral Responses to Noxious Thermal Stimulation in Chronic Low Back Pain Patients and Normal Controls. Neuroimage.

[15] Hsieh J-C, Ståhle-Bäckdahl M, Hägermark Ö (1996). Traumatic nociceptive pain activates the hypothalamus and the periaqueductal gray: a positron emission tomography study. Pain.

[16] Iadarola M (1998). Neural activation during acute capsaicin-evoked pain and allodynia assessed with PET. Brain.

[17] Petrovic P, Ingvar M, Stone-Elander S (1999). A PET activation study of dynamic mechanical allodynia in patients with mononeuropathy. Pain.

[18] Haas DC, Kent PF, Friedman DI (1993). Headache Caused by a Single Lesion of Multiple Sclerosis in the Periaqueductal Gray Area. Headache J Head Face Pain.

[19] Afridi S (2003). New onset migraine with a brain stem cavernous angioma. J Neurol Neurosurg Psychiatry.

[20] Goadsby P (2002). Neurovascular Headache and A Midbrain Vascular Malformation: Evidence for A Role of the Brainstem in Chronic Migraine. Cephalalgia.

[21] Burstein R, Yarnitsky D, Goor-Aryeh I (2000). An association between migraine and cutaneous allodynia. Ann Neurol.

[22] Lipton RB, Bigal ME, Ashina S (2008). Cutaneous allodynia in the migraine population. Ann Neurol.

[23] Burstein R, Collins B, Jakubowski M (2004). Defeating migraine pain with triptans: A race against the development of cutaneous allodynia. Ann Neurol.

[24] Pietrobon D, Moskowitz MA (2014). Chaos and commotion in the wake of cortical spreading depression and spreading depolarizations. Nat Rev Neurosci.

[25] Rasmussen BK, Olesen J (1992). Migraine With Aura and Migraine Without Aura: An Epidemiological Study. Cephalalgia.

[26] Hansen JM, Lipton RB, Dodick DW (2012). Migraine headache is present in the aura phase: A prospective study. Neurology.

[27] Olesen J, Larsen B, Lauritzen M (1981). Focal hyperemia followed by spreading oligemia and impaired activation of rcbf in classic migraine. Ann Neurol.

[28] Hadjikhani N, Sanchez del Rio M, Wu O (2001). Mechanisms of migraine aura revealed by functional MRI in human visual cortex. Proc Natl Acad Sci.

[29] Olesen J, Lauritzen M, Tfelt-Hansen P (1982). Spreading Cerebral Oligemia in Classical- and Normal Cerebral Blood Flow in Common Migraine. Headache J Head Face Pain.

[30] Aiba S, Tatsumoto M, Saisu A (2010). Prevalence of typical migraine aura without headache in Japanese ophthalmology clinics. Cephalalgia.

[31] Amos JF, Fleming JB (2000). Clinical description and review of migraine aura without headache. Optometry.

[32] Fisher CM (1986). Late-life migraine accompaniments–further experience. Stroke.

[33] Wijman CAC, Wolf PA, Kase CS (1998). Migrainous Visual Accompaniments Are Not Rare in Late Life. Stroke.

[34] Hansen JM, Hauge AW, Olesen J (2010). Calcitonin gene-related peptide triggers migraine-like attacks in patients with migraine with aura. Cephalalgia.

[35] Wiggers A, Ashina H, Hadjikhani N (2022). Brain barriers and their potential role in migraine pathophysiology. J Headache Pain.

[36] Amin FM, Hougaard A, Cramer SP (2017). Intact blood−brain barrier during spontaneous attacks of migraine without aura: a 3T DCE-MRI study. Eur J Neurol.

[37] Hougaard A, Amin FM, Christensen CE (2017). Increased brainstem perfusion, but no blood-brain barrier disruption, during attacks of migraine with aura. Brain.

[38] Donnelly A, Sinnott B, Boyle R, et al. Beware the middle-aged migraine: internal carotid artery dissection mimicking migraine in the emergency department. BMJ Case Rep. 2017;bcr-2017–221774. 10.1136/bcr-2017-221774 PMC5720261 29184007

[39] Ramadan NM, Tietjen GE, Levine SR (1991). Scintillating scotomata associated with internal carotid artery dissection: Report of three cases. Neurology.

[40] Thomsen AV, Sørensen MT, Ashina M (2021). Symptomatic migraine: a systematic review to establish a clinically important diagnostic entity. Headache J Head Face Pain.

[41] van den Maagdenberg AMJ, Pietrobon D, Pizzorusso T (2004). A cacna1a knockin migraine mouse model with increased susceptibility to cortical spreading depression. Neuron.

[42] Eikermann-Haerter K, Dileköz E, Kudo C, et al. Genetic and hormonal factors modulate spreading depression and transient hemiparesis in mouse models of familial hemiplegic migraine type 1. J Clin Invest. Epub ahead of print 22 December 2008. 10.1172/JCI36059. 10.1172/JCI36059 PMC2613474 19104150

[43] van den Maagdenberg AMJM, Pizzorusso T, Kaja S (2010). High cortical spreading depression susceptibility and migraine-associated symptoms in Ca v 2.1 S218L mice. Ann Neurol.

[44] Leo L, Gherardini L, Barone V (2011). Increased susceptibility to cortical spreading depression in the mouse model of familial hemiplegic migraine type 2. PLoS Genet.

[45] Nyholt DR, LaForge KS, Kallela M (2008). A high-density association screen of 155 ion transport genes for involvement with common migraine. Hum Mol Genet.

[46] Kirchmann M, Thomsen LL, Olesen J (2006). The CACNA1A and ATP1A2 genes are not involved in dominantly inherited migraine with aura. Am J Med Genet Part B Neuropsychiatr Genet.

[47] Jen JC, Kim GW, Dudding KA (2004). No Mutations in CACNA1A and ATP1A2 in Probands With Common Types of Migraine. Arch Neurol.

[48] Wieser T, Mueller C, Evers S, et al. Absence of Known Familial Hemiplegic Migraine (FHM) Mutations in the CACNA1A Gene in Patients with common Migraine: Implications for Genetic Testing. Clin Chem Lab Med; 41. Epub ahead of print 24 January 2003. 10.1515/CCLM.2003.042. 10.1515/CCLM.2003.042 12705332

[49] Younis S, Do TP, Ashina M (2021) Human Models. In: Maassen van den Brink, A., Martelletti P. (eds) Monoclonal Antibodies in Headache . Headache. Springer, Cham. 10.1007/978-3-030-69032-8_5

[50] Ghanizada H, Al-Karagholi MA-M, Arngrim N (2020). PACAP27 induces migraine-like attacks in migraine patients. Cephalalgia.

[51] Amin FM, Hougaard A, Schytz HW (2014). Investigation of the pathophysiological mechanisms of migraine attacks induced by pituitary adenylate cyclase-activating polypeptide-38. Brain.

[52] Pecile A, Guidobono F, Netti C (1987). Calcitonin gene-related peptide: antinociceptive activity in rats, comparison with calcitonin. Regul Pept.

[53] Candeletti S, Ferri S (1990). Antinociceptive profile of intracerebroventricular salmon calcitonin and calcitonin gene-related peptide in the mouse formalin test. Neuropeptides.

[54] Zhang YZ, Sjőlund B, Moller K (1993). Pituitary adenylate cyclase activating peptide produces a marked and long-lasting depression of a C-fibre-evoked flexion reflex. Neuroscience.

[55] Yamamoto T, Tatsuno I (1995). Antinociceptive effect of intrathecally administered pituitary adenylate cyclase activating polypeptide (PACAP) on the rat formalin test. Neurosci Lett.

[56] Fontaine D, Almairac F, Santucci S (2018). Dural and pial pain-sensitive structures in humans: new inputs from awake craniotomies. Brain.

[57] Al-Karagholi MA-M, Ghanizada H, Nielsen CAW, et al. Opening of ATP sensitive potassium channels causes migraine attacks with aura. Brain. Epub ahead of print March 2021. 10.1093/brain/awab136. 10.1093/brain/awab136 33768245

[58] Al-Karagholi MA-M, Ghanizada H, Waldorff Nielsen CA, et al. Opening of BKCa channels causes migraine attacks. Pain; Publish Ah. Epub ahead of print 16 March 2021. 10.1097/j.pain.0000000000002238. 10.1097/j.pain.0000000000002238 34252916

[59] Al-Karagholi MA-M, Ghanizada H, Hansen JM (2019). Extracranial activation of ATP-sensitive potassium channels induces vasodilation without nociceptive effects. Cephalalgia.

[60] Kullmann DM, Waxman SG (2010). Neurological channelopathies: new insights into disease mechanisms and ion channel function. J Physiol.

[61] Kremeyer B, Lopera F, Cox JJ (2010). A Gain-of-Function Mutation in TRPA1 Causes Familial Episodic Pain Syndrome. Neuron.

[62] Hautakangas H, Winsvold BS, Ruotsalainen SE (2022). Genome-wide analysis of 102,084 migraine cases identifies 123 risk loci and subtype-specific risk alleles. Nat Genet.

[63] Winsvold BS, Nelson CP, Malik R (2015). Genetic analysis for a shared biological basis between migraine and coronary artery disease. Neurol Genet.

[64] Malik R, Freilinger T, Winsvold BS (2015). Shared genetic basis for migraine and ischemic stroke: a genome-wide analysis of common variants. Neurology.

[65] Sacco S, Ornello R, Ripa P (2015). Migraine and risk of ischaemic heart disease: a systematic review and meta-analysis of observational studies. Eur J Neurol.

[66] Schurks M, Rist PM, Bigal ME (2009). Migraine and cardiovascular disease: systematic review and meta-analysis. BMJ.

[67] Mahmoud AN, Mentias A, Elgendy AY (2018). Migraine and the risk of cardiovascular and cerebrovascular events: a meta-analysis of 16 cohort studies including 1 152 407 subjects. BMJ Open.

[68] Dichgans M, Mayer M, Uttner I (1998). The phenotypic spectrum of CADASIL: Clinical findings in 102 cases. Ann Neurol.

[69] Montagna P, Gallassi R, Medori R (1988). MELAS syndrome: Characteristic migrainous and epileptic features and maternal transmission. Neurology.

[70] Ohama E, Ohara S, Ikuta F (1987). Mitochondrial angiopathy in cerebral blood vessels of mitochondrial eneephalomyopathy. Acta Neuropathol.

[71] Ashina M, Buse DC, Ashina H (2021). Migraine: integrated approaches to clinical management and emerging treatments. Lancet.

[72] Ashina M, Goadsby PJ, Dodick DW (2022). Assessment of Erenumab Safety and Efficacy in Patients With Migraine With and Without Aura. JAMA Neurol.

[73] Ashina M, McAllister P, Cady R, Hirman J, Ettrup A (2022) Efficacy and safety of eptinezumab in patients with migraine and self-reported aura: Post hoc analysis of PROMISE-1 and PROMISE-2. Cephalalgia 42(8):696–704. 10.1177/03331024221077646. Epub 2022 Mar 18 10.1177/03331024221077646 PMC9218409 35302389

[74] Burstein R, Blumenfeld AM, Silberstein SD (2020). Mechanism of Action of OnabotulinumtoxinA in Chronic Migraine: A Narrative Review. Headache J Head Face Pain.

[75] Gfrerer L, Xu W, Austen W (2022). OnabotulinumtoxinA alters inflammatory gene expression and immune cells in chronic headache patients. Brain.

[76] Lucioni A, Bales GT, Lotan TL (2008). Botulinum toxin type A inhibits sensory neuropeptide release in rat bladder models of acute injury and chronic inflammation. BJU Int.

[77] Durham PL, Cady R, Cady R (2004). Regulation of Calcitonin Gene-Related Peptide Secretion From Trigeminal Nerve Cells by Botulinum Toxin Type A: Implications for Migraine Therapy. Headache J Head Face Pain.

[78] Schankin CJ, Maniyar FH, Seo Y (2016). Ictal lack of binding to brain parenchyma suggests integrity of the blood–brain barrier for 11 C-dihydroergotamine during glyceryl trinitrate-induced migraine. Brain.

[79] Levy D, Jakubowski M, Burstein R (2004). Disruption of communication between peripheral and central trigeminovascular neurons mediates the antimigraine action of 5HT 1B/1D receptor agonists. Proc Natl Acad Sci.

[80] Khan S, Mohammad Amin F, Emil Christensen C (2019). Meningeal contribution to migraine pain: a magnetic resonance angiography study. Brain.

[81] Laurell K, Artto V, Bendtsen L (2016). Premonitory symptoms in migraine: A cross-sectional study in 2714 persons. Cephalalgia.

[82] Giffin NJ, Ruggiero L, Lipton RB (2003). Premonitory symptoms in migraine: An electronic diary study. Neurology.

[83] Maniyar FH, Sprenger T, Monteith T (2014). Brain activations in the premonitory phase of nitroglycerin-triggered migraine attacks. Brain.

[84] Schulte LH, Mehnert J, May A (2020). Longitudinal Neuroimaging over 30 Days: Temporal Characteristics of Migraine. Ann Neurol.

[85] Russell MB, Olesen J (1996). A nosographic analysis of the migraine aura in a general population. Brain.

[86] Lashley KS (1941). Patterns, of cerebral integration indicated by the scotomas of migraine. Arch Neurol Psychiatry.

[87] Ayata C, Lauritzen M (2015). Spreading Depression, Spreading Depolarizations, and the Cerebral Vasculature. Physiol Rev.

[88] Olesen J, Friberg L, Olsen TS (1990). Timing and topography of cerebral blood flow, aura, and headache during migraine attacks. Ann Neurol.

[89] Hadjikhani N, Albrecht DS, Mainero C (2020). Extra-Axial Inflammatory Signal in Parameninges in Migraine with Visual Aura. Ann Neurol.

[90] Zhang X, Levy D, Noseda R (2010). Activation of Meningeal Nociceptors by Cortical Spreading Depression: Implications for Migraine with Aura. J Neurosci.

[91] Harriott AM, Chung DY, Uner A (2021). Optogenetic Spreading Depression Elicits Trigeminal Pain and Anxiety Behavior. Ann Neurol.

[92] de Boer I, Terwindt GM, van den Maagdenberg AMJM (2020). Genetics of migraine aura: an update. J Headache Pain.

[93] Nozari A, Dilekoz E, Sukhotinsky I (2010). Microemboli may link spreading depression, migraine aura, and patent foramen ovale. Ann Neurol.

[94] Kurth T, Chabriat H, Bousser M-G (2012). Migraine and stroke: a complex association with clinical implications. Lancet Neurol.

[95] Wienecke T, Olesen J, Ashina M (2011). Discrepancy between strong cephalic arterial dilatation and mild headache caused by prostaglandin D 2 (PGD 2). Cephalalgia.

[96] Ashina M, Tfelt-Hansen P, Dalgaard P (2011). Lack of correlation between vasodilatation and pharmacologically induced immediate headache in healthy subjects. Cephalalgia.

[97] Amin FM, Asghar MS, Hougaard A (2013). Magnetic resonance angiography of intracranial and extracranial arteries in patients with spontaneous migraine without aura: a cross-sectional study. Lancet Neurol.

[98] Deen M, Hougaard A, Hansen HD (2019). Association Between Sumatriptan Treatment During a Migraine Attack and Central 5-HT 1B Receptor Binding. JAMA Neurol.

[99] Rubio-Beltrán E, Labastida-Ramírez A, Villalón CM (2018). Is selective 5-HT1F receptor agonism an entity apart from that of the triptans in antimigraine therapy?. Pharmacol Ther.

[100] Labastida-Ramírez A, Rubio-Beltrán E, Haanes KA (2020). Lasmiditan inhibits calcitonin gene-related peptide release in the rodent trigeminovascular system. Pain.

[101] Felgenhauer K (1974). Protein size and cerebrospinal fluid composition. Klin Wochenschr.

[102] Al-Karagholi MA-M, Ghanizada H, Nielsen CAW, et al. Opening of ATP sensitive potassium channels causes migraine attacks with aura. Brain. Epub ahead of print 26 March 2021. 10.1093/brain/awab136. 10.1093/brain/awab136 33768245

[103] Goadsby PJ, Edvinsson L, Ekman R (1988). Release of vasoactive peptides in the extracerebral circulation of humans and the cat during activation of the trigeminovascular system. Ann Neurol.

